# Devonian–Mississippian magmatism related to extensional collapse in Svalbard: implications for radiating dyke swarms

**DOI:** 10.12688/openreseurope.18798.1

**Published:** 2024-12-12

**Authors:** Jean-Baptiste P. Koehl, Sebastian Tappe, Gillian R. Foulger, Ingrid M. Anell

**Affiliations:** 1 Earth and Planetary Sciences, McGill University Faculty of Science, Montreal, Québec, H3A 0E8, Canada; 2 Geosciences, Universitetet i Oslo Institutt for geofag, Oslo, Oslo, 0371, Norway; 3 Geosciences, UiT The Arctic University of Norway, Tromsø, Troms, 9037, Norway; 4 Earth Sciences, Durham University Department of Earth Sciences, Durham, England, DH1 3LE, UK

**Keywords:** Svalbard, Devonian, Mississippian, Cretaceous, Paleoproterozoic, Neoproterozoic, large igneous province, radiating dyke swarm, Kola-Dnieper Large Igneous Province, Yakutsk-Vilyui Large Igneous Province, High Arctic Large Igneous Province, Timanian Orogen, Caledonian Orogen, inheritance, post-orogenic collapse

## Abstract

**Background:**

Despite extensive studies of the Mesozoic–Cenozoic magmatic history of Svalbard, little has been done on the Paleozoic magmatism due to fewer available outcrops.

**Methods:**

2D seismic reflection data were used to study magmatic intrusions in the subsurface of eastern Svalbard.

**Results:**

This work presents seismic evidence for west-dipping, Middle Devonian–Mississippian sills in eastern Spitsbergen, Svalbard. The sills crosscut a late Neoproterozoic Timanian thrust system, which was reworked during Caledonian contraction. The sills are unconformably overlain by relatively undeformed Pennsylvanian–Mesozoic sedimentary rocks and crosscut by Cretaceous dykes of the High Arctic Large Igneous Province. The sills probably intruded along extensional fractures during post-Caledonian reactivation–overprinting of the late Neoproterozoic thrust system. Kimberlitic accessory minerals in exposed contemporaneous intrusions and the chemical composition of chromium spinel grains in Upper Triassic sedimentary rocks in Svalbard suggest that the Middle Devonian–Mississippian intrusions in eastern Spitsbergen show affinities with diamond-rich kimberlites in northwestern Russia. Overall, the sills were emplaced during a regional episode of extension-related Devonian–Carboniferous magmatism in the Northern Hemisphere including the Kola–Dnieper and Yakutsk–Vilyui large igneous provinces.

**Conclusions:**

This work presents the first evidence for extensive Middle Devonian–Mississippian magmatism in Svalbard. These intrusions may be part of the Kola–Dnieper Large Igneous Province and intruded parallel to preexisting, Proterozoic–early Paleozoic orogenic structures. Their strike is inconsistent with a source from a potential mantle plume center in the eastern Barents Sea. Thus, the radiating emplacement pattern of the magmatic intrusions of the Kola–Dnieper Large Igneous Province are not the product of plume-related uplift but of structural inheritance. A similar line of reasoning is successfully applied to intrusions of the Yakutsk–Vilyui and High Arctic large igneous provinces.

## Plain language summary

The study presents the first evidence for extensive magmatism in Svalbard in the late Paleozoic at ca. 390–330 Ma. The composition of the magmatic intrusions suggests they are part of a large magmatic province extending thousands of km from Ukraine in the south to the Ural Mountains in the northeast and Svalbard in the northwest. The magmatic rocks intruded along existing tectonic structures formed at ca. 650–550 Ma during the collision of two tectonic plates. This work documents the impact of preexisting structures on subsequent magmatic events, especially in determining preferential pathways for magma in the crust. A similar reasoning can be applied to other contemporaneous groups of intrusions in Russia and to nearby younger intrusions in the Arctic (Yakutsk–Vilyui Large Igneous Province and High Arctic Large Igneous Province), which appear to follow structures related to old plate-collision events. Thus, the apparently radiating pattern displayed by those groups of intrusions is generated by previous episodes of deformation of the crust rather than by uplift and fracturing of the crust around potential mantle plumes, which are focus points of upwelling of magma from the deep Earth interior at the core–mantle boundary.

## Introduction

**Figure 1.**
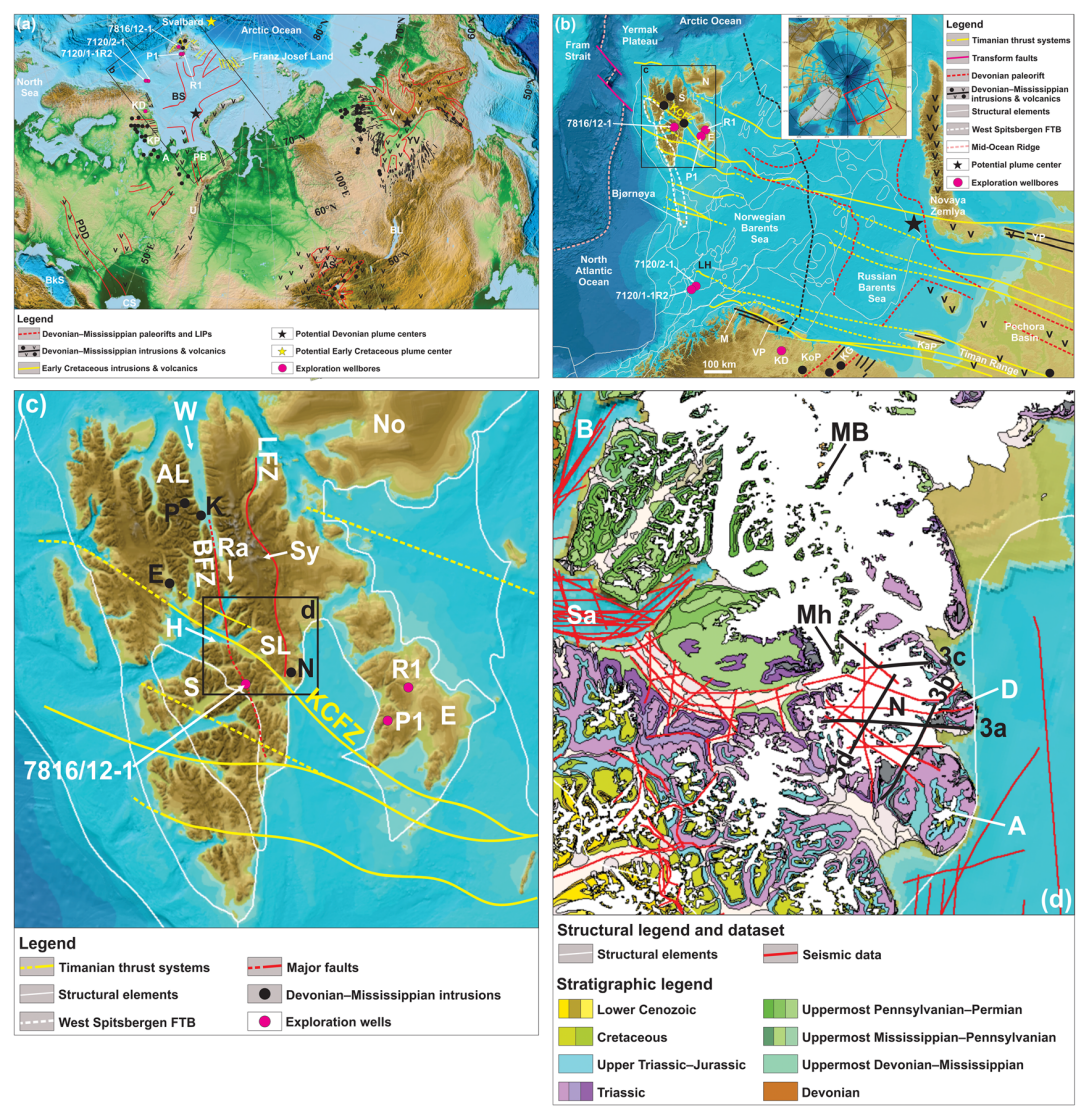
Overview maps of the study area and nearby magmatic provinces. ( **a**) Overview of the Devonian–Mississippian magmatic provinces discussed in the present study. The extent of the Barents Sea paleorift and Kola–Dnieper LIP is based on Puchkov *et al.* (2016), the Yakutsk–Vilyui LIP on Polyansky *et al.* (2017), and the Altay–Sayan LIP on Vorontsov *et al.* (2021). Basemap is from Amante and Eakins (2009). The potential Devonian–Mississippian plume centers in the Barents Sea and Yakutsk–Vilyui LIP are from Puchkov *et al.* (2016), Ernst and Buchan (1997), and Kiselev *et al.* (2012). The tentative Early Cretaceous plume center shows the potential intersection of Early Cretaceous intrusions north of Svalbard and Franz Josef Land. All inferred plume centers are shown in present-day coordinates. Abbreviations: A: Arkhangelsk Province; AS: Altay–Sayan LIP; BkS: Black Sea; BL: Baikal Lake; BS: Barents Sea paleorift; CS: Caspian Sea; KD: Kola super deep well; KP: Kola Peninsula; PB: Pechora Basin; PDD: Pripyat–Dnieper–Donets Rift; U: Urals; V: Verkhoyansk Mountains; YV: Yakutsk–Vilyui LIP. ( **b**) Overview map of the main structural elements in the Barents Sea region showing the strike and extent of Timanian thrust systems (yellow lines) based on Koehl *et al.* ( 2022a; 2023c), Koehl (2024), and Koehl and Stokmo (2024) and of the Devonian paleorift and related Devonian–Mississippian intrusions and volcanics (including kimberlites) based on Kramm *et al.* (1993) and Puchkov *et al.* (2016). The potential mantle plume center is from Puchkov *et al.* (2016). The structural elements in the Barents Sea (thin white lines) are from the Norwegian Offshore Directorate and the basemap from Jakobsson *et al.* (2012). Abbreviations: E: Edgeøya; KaP: Kanin Peninsula; KD: Kola super deep well; KCFZ: Kongsfjorden–Cowanodden fault zone; KG: Kontozero Graben; KoP: Kola Peninsula; LH: Loppa High; M: Magerøya; N: Nordaustlandet; P1: Plurdalen-1 exploration well; R1: Raddedalen-1 exploration well; S: Spitsbergen; VP: Varanger Peninsula; YP: Yugorsky Peninsula. ( **c**) Overview map of the Svalbard Archipelago. The Devonian–Mississippian intrusions are from Evdokimov *et al.* (2006) and Sirotkin *et al.* ( 2012; Krosspynten, Purpurdalen, and Ekmanfjorden) and the present study (Nordmannsfonna). Timanian thrust systems are from Koehl *et al.* ( 2022a; 2023b; 2023c) and Koehl (2024), and basemap is from Jakobsson *et al.* (2012). Abbreviations: AL: Andrée Land; BFZ: Billefjorden Fault Zone; E: Ekmanfjorden intrusions; Ed: Edgeøya; H: Hatten; K: Krosspynten intrusions; KCFZ: Kongsfjorden–Cowanodden fault zone; LFZ: Lomfjorden Fault Zone; N: Nordmannsfonna intrusions (present study); No: Nordaustlandet; P: Purpurdalen; P1: Plurdalen-1 exploration well; Ra: Ragnarbreen; R1: Raddedalen-1 exploration well; S: Spitsbergen; SL: Sabine Land; Sy: Systertoppane; W: Wijdefjorden. ( **d**) Geological map of the study area in Nordmannsfonna in eastern Spitsbergen. The stratigraphy and associated color scheme are from Dallmann (2015). Abbreviations: A: Agardhfjellet; B: Billefjorden; D: Domen; MB: Malte Brunfjellet; Mh: Moskushornet; N: Nordmannsfonna; Sa: Sassenfjorden.

The Devonian–Mississippian was characterized by particularly voluminous magmatism, creating several large igneous provinces (LIPs) in Eurasia including the Altay–Sayan, Yakutsk–Vilyui, and Kola–Dnieper LIPs ( Figure 1a). Among these, the Kola–Dnieper LIP is believed to extend from eastern Europe (Pripyat–Dnieper–Donets Rift) to the Barents Sea ( Figure 1a). In the Barents Sea, it consists of a broad paleorift and related intrusions located mainly in the Russian sector of the Barents Sea, but also in northern Norway. These follow preexisting, WNW–ESE- to NW–SE-striking, late Neoproterozoic Timanian orogenic structures and NNE–SSW- to NE–SW-striking, early–mid Paleozoic, Caledonian structures ( Figure 1b).

Little is known about the western extent of the paleorift and associated Kola–Dnieper LIP. A few studies reported intrusions at Krosspynten, Purpurdalen, and Ekmanfjorden in Svalbard showing affinities with those of the Kola–Dnieper LIP ( Evdokimov *et al.*, 2006; Sirotkin *et al.*, 2012; Figure 1a–c). Similarities included evidence of kimberlitic accessory minerals such as pyrope, chromium diopside, moissanite, and chromium spinel xenocrysts ( Evdokimov *et al.*, 2006).

Svalbard’s Mesozoic–Cenozoic magmatic history is relatively well constrained, and includes extensive Cretaceous dolerite dykes and sills associated with the High Arctic Large Igneous Province (HALIP; Birkenmajer & Morawski, 1960; Birkenmajer *et al.*, 2010; Burov *et al.*, 1977; Corfu *et al.*, 2013; Foulger *et al.*, 2024; Grogan *et al.*, 2000; Maher, 2001; Nejbert *et al.*, 2011; Parker, 1966; Senger *et al.*, 2013; Senger *et al.*, 2014). The Cenozoic was marked by the emplacement of Miocene tholeitic basalts and Pleistocene alkali basalts, which occur respectively in northern and northwestern Spitsbergen as lava flows and plugs ( Amundsen *et al.*, 1987; Burov, 1965; Gjelsvik, 1963; Griffin *et al.*, 2012; Hansen *et al.*, 2003; Senger *et al.*, 2023; Skjelkvåle *et al.*, 1989).

By contrast, the magmatic history of Svalbard during the Paleozoic is only poorly understood and includes the kimberlitic to lamprophyric intrusions at Krosspynten, Purpurdalen, and Ekmanfjorden ( Evdokimov *et al.*, 2006; Sirotkin *et al.*, 2012; Figure 1c), and a few intrusions in southern Spitsbergen ( Birkenmajer *et al.*, 2010). Fieldwork in northern Nordaustlandet in 2023 also revealed such rare lamprophyric dyke intrusives. A major challenge is the lack of suitable outcrops and the apparent scarcity of Paleozoic intrusions above sea level.

Late Paleozoic intrusions were reported in subsurface seismic reflection data in Nordmannsfonna in eastern Spitsbergen by Koehl *et al.* ( 2022a; Figure 1c–d) and more detailed results and analysis of these is presented herein. The study explores possible relationships of the intrusions to the geochemical signatures of minerals in Upper Triassic sedimentary rocks, and to those of coeval, kimberlitic and lamprophyric magmatic systems in other Arctic areas, including the Devonian–Mississippian Yakutsk–Vilyui ( Courtillot *et al.*, 2010; Hamilton *et al.*, 2004; Powerman *et al.*, 2013; Ricci *et al.*, 2013; Tretiakova *et al.*, 2017) and Kola–Dnieper LIPs in northern Russia and eastern Europe ( Larionova *et al.*, 2016; Puchkov *et al.*, 2016; Terekhov *et al.*, 2012).

The findings have implications for the tectono-magmatic history of Arctic regions including their mineral exploration potential, as well as for the geometry and extent of Devonian–Mississippian LIPs. This study shows that magmatism emplaced contemporaneously with flood basalts may extend great distances from the main LIP. Those distant magmas were sourced locally and their crustal paths mainly controlled by pre-existing lithospheric structures.

## Geological setting

### Pre-devonian deformation of basement rocks at Nordmannsfonna in Svalbard

In Nordmannsfonna (see Figure 1c–d for location), seismic reflection data show that basement rocks are deformed into a major, north- to NNE-plunging antiform, which has been interpreted as a Timanian thrust fault that was folded during the Caledonian Orogeny ( Koehl *et al.*, 2022a). The antiform is unconformably overlain by Pennsylvanian–Mesozoic sedimentary strata and offset by the Agardhbukta Fault. The latter is a probable segment of the Lomfjorden Fault Zone ( Andresen *et al.*, 1992; Piepjohn *et al.*, 2019), which formed as a Caledonian thrust, was later partly inverted during Devonian–Mississippian late–post-orogenic extension, and mildly reactivated as a Eurekan thrust in the early Cenozoic ( Koehl *et al.*, 2022a). Major strike-slip movement along the Lomfjorden Fault Zone is unlikely because of the contiguous nature of Timanian thrust systems across the northwestern Russia, the Barents Sea, and Svalbard, including key examples in Sassenfjorden, Billefjorden, and Nordmannsfonna in central and eastern Spitsbergen ( Koehl *et al.*, 2022a; Koehl *et al.*, 2023a; Koehl *et al.*, 2023b; Figure 1c–d).

### Devonian–carboniferous tectonic setting of Svalbard

The Svalbard Archipelago is thought to have had a complex tectonic history in the early Paleozoic up until the Late Devonian when the three basement terranes constituting the archipelago were supposedly accreted during the Caledonian and Svalbardian orogenies along large, N–S-striking fault zones such as the Lomfjorden and Billefjorden fault zones ( Harland, 1969; Harland *et al.*, 1974; Harland *et al.*, 1992; Piepjohn, 2000; Figure 1c). However, continuous Timanian (650–550 Ma) thrust systems extending from northwestern Russia to central and southwestern Spitsbergen ( Faehnrich *et al.*, 2020; Koehl *et al.*, 2022a; Majka *et al.*, 2008) and the western Barents Sea ( Koehl *et al.*, 2023c) suggest that the Svalbard Archipelago was already assembled during the late Neoproterozoic. In addition, recent studies suggest that the Late Devonian (–earliest Mississippian?) Svalbardian Orogeny did not occur in Svalbard due to numerous inconsistencies in the structural and paleontological record ( Berry & Marshall, 2015; Koehl *et al.*, 2022b; Lindemann *et al.*, 2013; Marshall *et al.*, 2015; Newman *et al.*, 2019; Newman *et al.*, 2020; Newman *et al.*, 2021; Scheibner *et al.*, 2012). These studies, together with new latest Silurian to latest Devonian geochronological ages for the Keisarhjelmen extensional detachment in northwestern Spitsbergen ( Braathen *et al.*, 2018), indicate that Svalbard underwent dominantly late–post-Caledonian extensional collapse during the Devonian.

In the latest Devonian, sedimentary rocks of the Billefjorden Group started to deposit during continued extension ( Aakvik, 1981; Gjelberg, 1984; McCann & Dallmann, 1996) as suggested by small growth strata in Odellfjellet ( Koehl & Muñoz-Barrera, 2018). Extension continued into the Pennsylvanian as indicated by the deposition of hundreds of meters of uppermost Mississippian to lowermost Permian sediments of the Gipsdalen Group in the Billefjorden Trough ( Braathen *et al.*, 2011; Cutbill & Challinor, 1965; Gjelberg & Steel, 1981; Pickard *et al.*, 1996; Smyrak-Sikora *et al.*, 2019). It started to decline in the Early–Middle Pennsylvanian ( Dallmann, 1993; Koehl & Muñoz-Barrera, 2018; Koehl *et al.*, 2023b; Koehl *et al.*, 2023d).

In the study area, palynological data for rocks of the Malte Brunfjellet Formation yielded a mid–late Early Pennsylvanian age ( Scheibner *et al.*, 2015). At Malte Brunfjellet, these rocks overlie unconformably metamorphosed Neoproterozoic basement rocks and are overlain conformably by uppermost Pennsylvanian–earliest Permian carbonate-dominated sedimentary rocks of the Wordiekammen Formation ( Dallmann *et al.*, 2009; Dallmann, 2015; Scheibner *et al.*, 2015). Farther south, the latter are overlain conformably by lower Permian rocks of the Gipshuken and Kapp Starostin formations ( Andresen *et al.*, 1992; Cutbill & Challinor, 1965; Dallmann *et al.*, 1999; Dallmann *et al.*, 2009; Dallmann, 2015; Haremo & Andresen, 1992).

### Devonian–Mississippian magmatism in Siberia, Baltica, and Svalbard

The Devonian–Mississippian was marked by intense magmatism and the formation of LIPs in Siberia and Baltica, the Yakutsk–Vilyui LIP and the Kola–Dnieper LIP respectively ( Ernst *et al.*, 2020; Figure 1a). A few related intrusions are also reported from Svalbard ( Figure 1a–b), which was part of Baltica from at least the late Neoproterozoic (ca. 650 Ma; Koehl *et al.*, 2022a; Koehl *et al.*, 2023c).

***Siberia***

**Yakutsk–Vilyui LIP**

The Yakutsk–Vilyui LIP ( Figure 1a) comprises low-magnesium basaltic volcanic and intrusive rocks as well as high-magnesium kimberlites and related rocks. K–Ar, Ar–Ar, Sm–Nd, Lu–Hf, and U–Pb geochronology indicate that the LIP intruded the Siberian Craton during the Late Devonian–Mississippian at 381–338 Ma ( Courtillot *et al.*, 2010; Hamilton *et al.*, 2004; Powerman *et al.*, 2013; Ricci *et al.*, 2013; Sun *et al.*, 2014; Sun *et al.*, 2018; Tappe *et al.*, 2018; Tretiakova *et al.*, 2017). The LIP is associated with evaporitic sedimentary basins up to 6 km thick bounded by normal faults with both ENE–WSW and WNW–ESE strikes that formed during extension ( Danukalova *et al.*, 2014; Ershova *et al.*, 2014; Polyansky *et al.*, 2013; Polyansky *et al.*, 2017). The LIP has been interpreted to reflect either rift- and plume-related magmatism ( Kiselev *et al.*, 2006; Kiselev *et al.*, 2012; Puchkov *et al.*, 2016) or magmatism at an active continental margin ( Ivanov, 2015). Nevertheless, the radiating (triple junction-like) geometry of the dyke swarms and rift basins ( Kiselev *et al.*, 2012) have been interpreted as supporting a plume origin.

**Altay–Sayan region**

The Altay–Sayan region at the southwestern margin of the Siberian Craton ( Figure 1a) is characterized by large volumes of gabbroic and granitic intrusions ( Predtechensky & Krasnov, 1967; Vorontsov *et al.*, 2013). Magmatism occurred in two episodes during the Early–Middle Devonian (408–393 Ma) and the Late Devonian–Mississippian ( Babin *et al.*, 2004; Kuzmin *et al.*, 2010; Malkovets *et al.*, 2003; Predtechensky & Krasnov, 1967; Vorontsov *et al.*, 2021). The geochemical compositions of the intrusive rocks have been variously claimed to be related to plume, subduction, and backarc extensional ( Broussolle *et al.*, 2019; Kong *et al.*, 2023; Safonova *et al.*, 2004; Vorontsov *et al.*, 2013). This illustrates the current debate around the current understanding of these magmatic provinces.

***Baltica***

**Kola–Dnieper LIP**

Pripyat–Dnieper–Donets Rift

 The Pripyat–Dnieper–Donets Rift in eastern Europe is a 2000 kilometers long, WNW–ESE-striking graben or aulacogen that extends from the northeastern edge of the Azov Sea in the southeast to Belarus in the northwest, crosscutting Precambrian rocks of the East European Craton ( Chekunov *et al.*, 1992; Stovba *et al.*, 1996; Wilson & Lyashkevich, 1996; Figure 1a). It comprises several basins filled with up to 4 km thick Middle Devonian–Mississippian volcaniclastic and evaporitic successions, such as the Pripyat and Donets troughs ( Chekunov *et al.*, 1992; Stephenson *et al.*, 1993; Stovba *et al.*, 1996; Wilson & Lyashkevich, 1996). Ultramafic–mafic rocks such as alkaline basalts dominate the magmatic record and were emplaced both as lava flows, sills, and dykes ( Chekunov *et al.*, 1992; Starostenko *et al.*, 2018; Stephenson *et al.*, 1993; Wilson & Lyashkevich, 1996). Interpretations for these magmas include a backarc origin related to the onset of Variscan subduction in central Europe, rifting, and a cluster of mantle plume fingers ( Chekunov *et al.*, 1992; Sazonova *et al.*, 2019; Starostenko *et al.*, 2018; Wilson & Lyashkevich, 1996).

Northwestern Russia

In northwestern Russia, Devonian–Mississippian magmatism was widespread and the igneous products are of high economic importance because of the occurrence of critical minerals and diamonds. On the Kola Peninsula, it includes ultramafic-carbonatite complexes of the Kola Alkaline Province ( Downes *et al.*, 2005), which intruded Neoproterozoic metasedimentary rocks during the formation of the ENE–WSW-striking Kontozero Graben and associated deposition of lacustrine sediments in the Middle Devonian–Early Mississippian ( Kramm *et al.*, 1993). In the Arkhangelsk Alkaline Igneous Province, kimberlites and lamprophyric rocks yielded Late Devonian ages ( Kargin *et al.*, 2021; Larionova *et al.*, 2016; Mahotkin *et al.*, 2000).

In the Russian sector of the Barents Sea, Middle Devonian–Mississippian extension resulted in the development of a complex network of basins and highs, some of which can be traced onshore to the Pechora Basin, the Timan Range, the Kanin Peninsula, and Novaya Zemlya where they display a prominent WNW–ESE strike ( Baturin *et al.*, 1991; Klimenko *et al.*, 2011; Lopatin *et al.*, 2001; O’Leary *et al.*, 2004; Pease *et al.*, 2016; Wilson *et al.*, 1999; Figure 1a). Onshore igneous rocks (including those from the Kola super-deep well) comprise dominantly dolerite sills and layered basalts and tuffs of Middle Devonian to Early Mississippian ages ( Lopatin *et al.*, 2001; Pease *et al.*, 2016; Wilson *et al.*, 1999).

Possible causes of Devonian–Mississippian rifting and magmatism in northwestern Russia are backarc extension related to far-field effects from subduction in the Urals and/or post-Caledonian extension ( Nikishin *et al.*, 1996; O’Leary *et al.*, 2004; Pease *et al.*, 2016; Ziegler, 1988; Zonenshain *et al.*, 1990). A mantle plume has also been proposed ( Ernst *et al.*, 2020; Puchkov *et al.*, 2016). Convergence of several Devonian dyke swarms towards a focal point in the Russian sector of the Barents Sea have been cited in support of a mantle plume origin for this LIP-style magmatism ( Puchkov *et al.*, 2016).

Northern Norway (‘mainland’)

 In northern Norway, Middle Devonian–Mississippian magmatism related to the Kola–Dnieper LIP may include N–S- and NE–SW-striking dolerite dykes on Varanger Peninsula and WNW–ESE-striking in Magerøya and adjacent areas ( Figure 1b). These dyke intrusions appear clearly on regional high-resolution magnetic data and coincide with narrow positive anomalies ( Koehl *et al.*, 2019; Nasuti *et al.*, 2015). Offshore, they can be traced by high-amplitude seismic reflections ( Koehl & Stokmo, 2024). The dolerite dykes in Varanger Peninsula yielded Middle–Late Devonian ages ( Guise & Roberts, 2002; Roberts, 2011; Roberts & Walker, 1996; Roberts & Walker, 1997), whereas Mississippian ages were obtained for the dolerite dykes in Magerøya ( Lippard & Prestvik, 1995; Lippard & Prestvik, 1997; Roberts *et al.* 1991).

**Svalbard**

**Figure 2.**
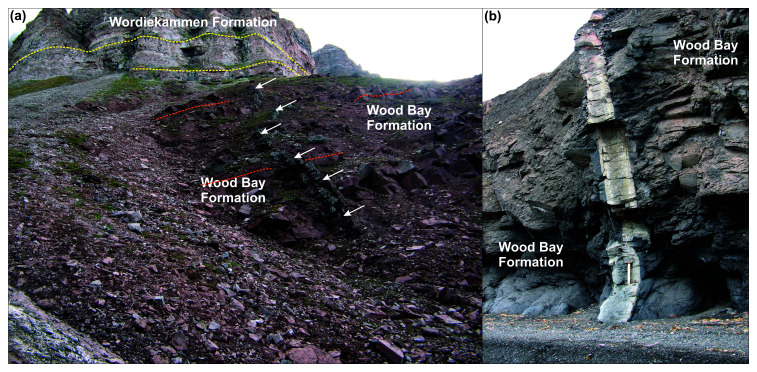
Outcrop photographs of Devonian–Mississippian dykes in ( **a**) Ekmanfjorden and ( **b**) Krosspynten. The basaltic dyke in ( **a**) is depicted by white arrows and terminates below the angular unconformity between gently dipping reddish sedimentary rocks of the Lower Devonian Wood Bay Formation with flat-lying carbonate-dominated strata of the uppermost Pennsylvanian–lowermost Permian Wordiekammen Formation. The photos are from Alexander Sirotkin.

In James I Land in central Spitsbergen, a few sub-vertical to moderately dipping serpentinized basaltic dykes intrude Lower Devonian sedimentary strata of the Wood Bay Formation ( Evdokimov *et al.*, 2006; Sirotkin *et al.*, 2012). One of these dykes is clearly truncated by an erosional unconformity and overlain by presumably Middle Devonian carbonates ( Evdokimov *et al.*, 2006). The only carbonate-bearing Devonian rock unit in Spitsbergen, the Skamdalen Member of the Grey Hoek Formation, crops out in Andrée Land in northern Spitsbergen ( Dallmann, 2015; Dallmann *et al.*, 2005; Dallmann *et al.*, 2006; Dallmann *et al.*, 2010). In addition, field observations indicate that the overlying carbonate beds are not Devonian but Carboniferous, specifically uppermost Pennsylvanian–lowermost Permian carbonates of the Wordiekammen Formation ( Figure 2a). Thus, the basaltic dykes in Ekmanfjorden intruded in the Middle Devonian–early Late Pennsylvanian.

At Krosspynten in Andrée Land ( Figure 1c), dozens of dominantly NNW–SSE-trending, up to 4 m-thick picritic dykes crosscut Lower Devonian sedimentary rocks of the Wood Bay Formation and yielded robust Middle Mississippian to Early Pennsylvanian Rb–Sr isochron ages and less reliable latest Silurian–earliest Permian K–Ar ages ( Evdokimov *et al.*, 2006). These dykes were previously referred to as monchiquites, a glassy alkaline lamprophyre variety ( Gayer *et al.*, 1966). They contain chromium spinel and mantle-derived peridotite xenoliths up to 3 cm in diameter indicating a primitive character and relatively deep origin from the upper mantle. These dykes are mildly folded ( Figure 2b) probably as a result of Eurekan contraction. Similar minor intrusions were also observed in Systertoppane and Ragnarbreen (see Figure 1c for location), where they truncate late–post-Caledonian granite ( Teben’kov *et al.*, 1996).

Other occurrences of Devonian–Mississippian dyke intrusions were reported from central Nordaustlandet and southern Spitsbergen. In Nordaustlandet, 2–5 m thick, 50–100 m long, stepping lamprophyre dykes truncate Caledonian basement rocks ( Ohta, 1982). These dykes yielded Late Devonian–Early Mississippian K–Ar ages (376–362 Ma; Krasil’scikov, 1973; Ohta, 1982). In southern Spitsbergen, Birkenmajer *et al.* (2010) obtained Mississippian and Early Devonian K–Ar ages for a metadolerite dyke and hornblende in a gabbroic intrusion at Asbestodden respectively. The Early Devonian age is probably a minimum age as suggested by excess argon in the sample. In addition, their study also includes an Early Permian (whole rock) age for the gabbroic intrusion, which may reflect overprinting during Mesozoic hydrothermal alteration.

### Cretaceous diabasodden suite in Svalbard

 Cretaceous igneous rocks of the HALIP occur regionally as the Diabasodden Suite in Svalbard ( Figure 1d; Dallmann *et al.*, 1999; Grogan *et al.*, 2000; Maher, 2001; Nejbert *et al.*, 2011; Senger *et al.*, 2013). They are characterized by dominant meter- to hundred-meter-thick sills in Pennsylvanian–Mesozoic sedimentary strata ( Maher, 2001; Nejbert *et al.*, 2011; Senger *et al.*, 2013) and subsidiary dykes mostly occurring in metamorphosed basement rocks ( Birkenmajer, 1986; Birkenmajer & Morawski, 1960; Birkenmajer *et al.*, 2010; Frebold, 1935). In the study area, the intrusive bodies are hosted by Pennsylvanian–Mesozoic sedimentary rocks ( Dallmann, 2015; Senger *et al.*, 2013). Both the sills and dykes are still in their original emplacement positions (i.e., subhorizontal and subvertical), e.g., the Hatten feeder dyke ( Nejbert *et al.*, 2011; Senger *et al.*, 2013), since they were intruded some distance from the main early Cenozoic Eurekan deformation, which shaped the West Spitsbergen Fold-and-Thrust Belt in western Spitsbergen ( Figure 1b).

The Diabasodden Suite in Svalbard comprises three major axes of outcrops following major structural trends. In western to southern Spitsbergen and in eastern Spitsbergen to Nordaustlandet, outcrops of early Cretaceous intrusions are found along NNW–SSE-trending axes ( Dallmann, 2015; Maher, 2001), i.e., parallel to the Caledonian and Grenvillian tectonic trends (e.g., Bernard-Griffiths *et al.*, 1993; Dallmeyer *et al.*, 1990; Dumais & Brönner, 2020; Gee *et al.*, 1994; Johansson *et al.*, 2004; Johansson *et al.*, 2005; Witt-Nilsson *et al.*, 1998), whereas they occur along a WNW–ESE-trending axis in central Spitsbergen ( Dallmann, 2015; Maher, 2001; Senger *et al.*, 2013), i.e., parallel to late Neoproterozoic Timanian fabrics and faults ( Koehl *et al.*, 2022a; Koehl *et al.*, 2023b; Figure 1b–c).

### Eurekan deformation in eastern Spitsbergen

During the early Cenozoic, Svalbard and Greenland collided, which resulted in formation of the West Spitsbergen Fold-and-Thrust Belt in western Spitsbergen ( Bergh & Andresen, 1990; Bergh *et al.*, 2000; Birkenmajer, 1972; Dallmann, 1988; Dallmann & Maher, 1989; Dallmann *et al.*, 1993; Harland, 1969; Maher, 1988; Maher *et al.*, 1986; Tessensohn, 2001; Welbon & Maher, 1992) and gradually milder deformation in central and eastern Svalbard ( Andresen *et al.*, 1992; Haremo & Andresen, 1992; Koehl, 2021; Koehl *et al.*, 2022a). Most early Cenozoic Eurekan structures in eastern Spitsbergen are gently dipping to sub-horizontal deformation zones and décollements ( Andresen *et al.*, 1992; Haremo & Andresen, 1992).

In addition, minor early Cenozoic, Eurekan, top-west reverse faulting occurred along the Agardhbukta Fault in Nordmannsfonna ( Koehl *et al.*, 2022a). Other workers have argued for major strike-slip movements along the Agardhbukta Fault segment of the Lomfjorden Fault Zone ( Piepjohn *et al.*, 2019). However, this proposition is in conflict with the absence of lateral offset of the crustal-scale late Neoproterozoic Timanian thrust system across the Agardhbukta Fault in Nordmannsfonna ( Koehl *et al.*, 2022a).

## Methods

We used 2D Two-Way Time (TWT) seismic reflection data from the Norwegian National Data Repository for Petroleum Data ( DISKOS database – survey ST9201) analyzed in Petrel (version 2021.3) to study a sub-surface magmatic system in Nordmannsfonna, eastern Spitsbergen. An alternative open-source software is OpendTect. Where possible, the seismic interpretation was tied to onshore geological maps produced by the Norwegian Polar Institute and previous studies, which show outcrops of Pennsylvanian to lower Cretaceous sedimentary rocks and Cretaceous dolerite intrusions ( Andresen *et al.*, 1992; Dallmann, 2015; Haremo & Andresen, 1992). Notably, Cretaceous dolerite of the Diabasodden Suite at Domen and Agardhfjellet and Pennsylvanian–Permian sedimentary rocks of the Gipsdalen Group at Moskushornet provide robust ties to the interpreted seismic data (svalbardkartet.npolar.no; Figure 1d). Time constraints exist for Pennsylvanian–Permian sedimentary rocks at Malte Brunfjellet, where an Early Pennsylvanian age was obtained based on foraminifer biostratigraphy and cyclostratigraphy ( Scheibner *et al.*, 2015).

**Figure 3.**
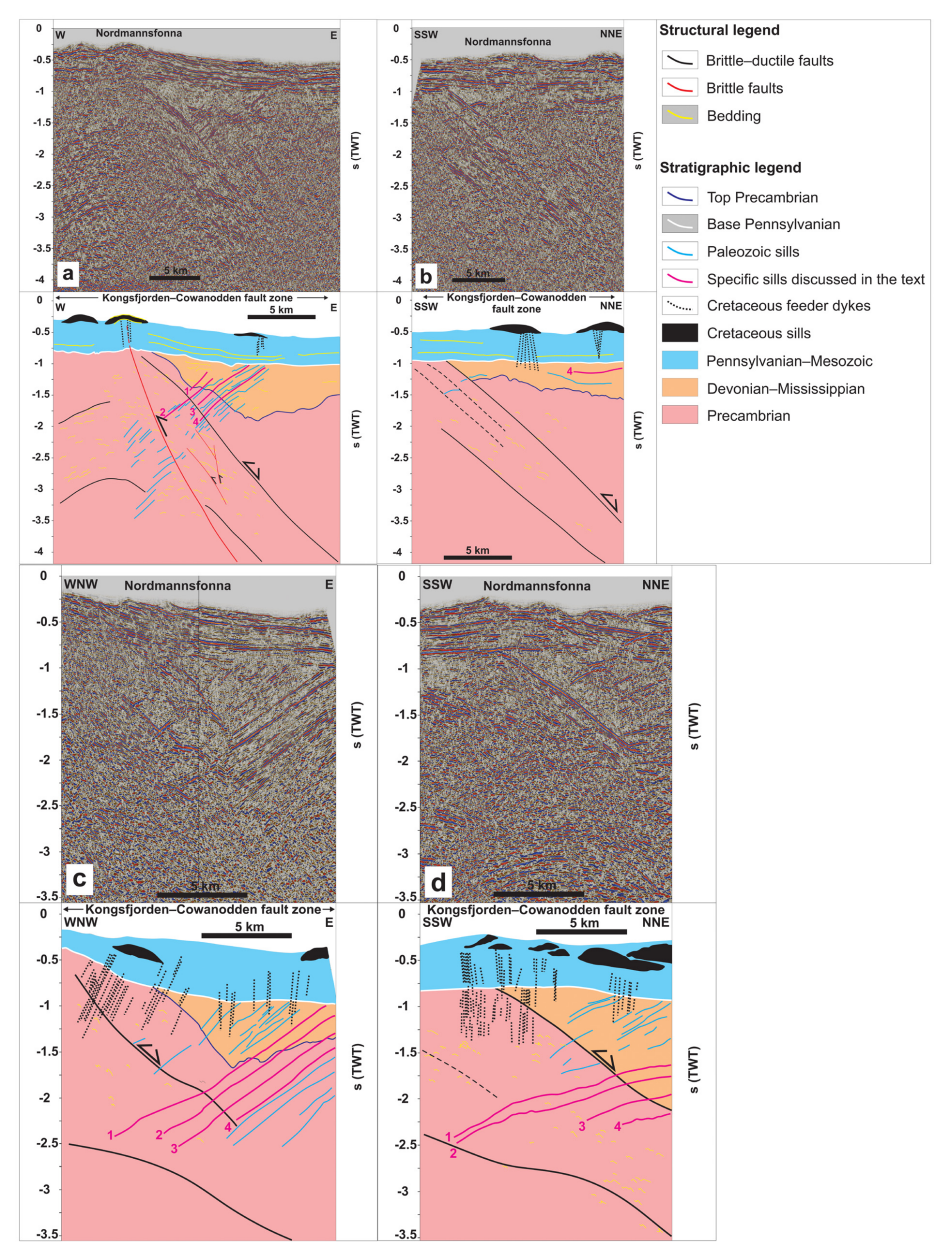
( **a–d**) Interpreted seismic sections in Nordmannsfonna, eastern Spitsbergen. The seismic profiles show moderately west-dipping, Middle Devonian–Mississippian sills and Cretaceous intrusions related to the HALIP. The profiles also show the Kongsfjorden–Cowanodden fault zone, which formed as a top-SSW Timanian thrust in the late Neoproterozoic and was reactivated and overprinted during the early–mid Paleozoic Caledonian Orogeny, late–post-Caledonian extensional collapse in the Devonian–Carboniferous, and early Cenozoic Eurekan contraction. ( **a**– **b**) are modified after Koehl *et al.* (2022a).

We identified a few Cretaceous intrusion centers on the seismic data (black-shaded in Figure 3a–d) and the various ties to exploration wells and onshore outcrops allowed segregation of Early Cretaceous intrusions from older ones. Basaltic intrusions generally display high acoustic impedance contrasts with surrounding sedimentary strata and therefore appear as high-amplitude reflections. In contrast, felsic intrusions do not resolve well due to lower density and sonic velocity, thus resulting in lower acoustic impedance contrast ( Mark *et al.*, 2018; Phillips *et al.*, 2018a). Bedding surfaces are generally truncated or terminate as onlap, toplap, or downlap reflections, whereas intrusions truncate other reflections though without offset.

**Figure 4.**
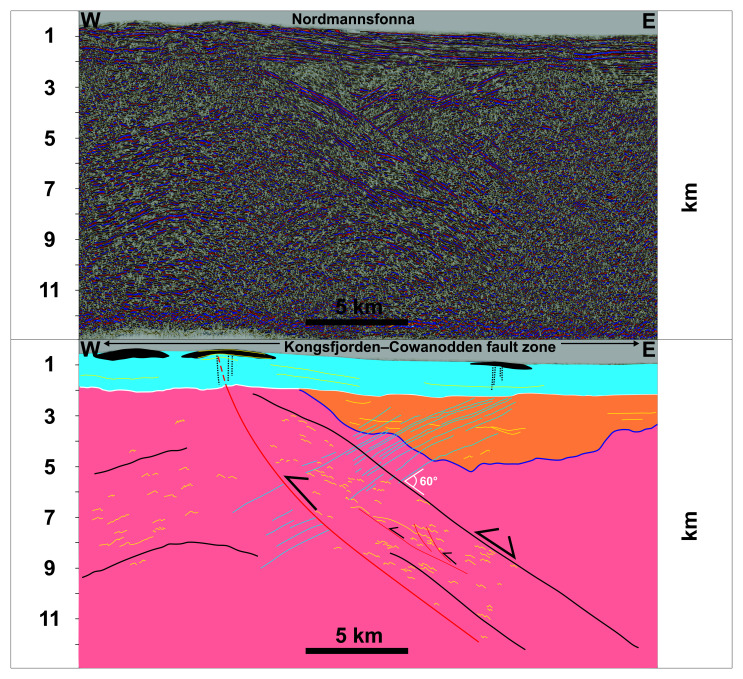
Depth-converted seismic section of Figure 3a. The section shows moderately west-dipping geometry of the sills and their intrusion at c. 60° angle off the Kongsfjorden–Cowanodden fault zone at Nordmannsfonna.

One E–W-trending seismic line was depth-converted to illustrate better the west-dipping geometry of the intrusions ( Figure 4). To do this, the seismic velocities from Gernigon *et al.* (2018) were used. The seismic data was also tied to the Plurdalen-1 and Raddedalen-1 well bores in Edgeøya ( Bro & Shvarts, 1983; Harland & Kelly, 1997; Figure 1b–c). The present study favored the interpretation of Ordovician–Silurian rocks by Bro and Shvarts (1983) in the Raddedalen-1 well (see Section 3 in Koehl *et al.*, 2022a for discussion on the well data). The Plurdalen-1 well bore penetrated Lower Devonian red beds dated to ca. 410 Ma ( Harland & Kelly, 1997, their figure 5.14).

The Base Pennsylvanian unconformity in Nordmannsfonna was interpreted as a major unconformity truncating both Precambrian rocks and basement-seated structures (e.g., folded Kongsfjorden–Cowanodden fault zone) and Devonian–Mississippian sedimentary rocks and intrusions. This choice is supported by the strong dominance of unconformable contacts between Devonian–Mississippian sedimentary rocks (Red Bay, Andrée Land, and Billefjorden groups) and uppermost Mississippian–Pennsylvanian deposits (Gipsdalen Group) in Svalbard, the Barents Sea, and adjacent Arctic areas ( Bugge *et al.*, 1995; Cutbill & Challinor, 1965; Cutbill *et al.*, 1976; Dalhoff *et al.*, 2000; Dalhoff & Stemmerik, 2000; Gjelberg, 1981; Koehl *et al.*, 2018; Koehl *et al.*, 2023b; Koehl & Muñoz-Barrera, 2018; Larssen *et al.*, 2005; Stemmerik *et al.*, 1991; Stemmerik *et al.*, 1998; Stemmerik, 2000; Worsley *et al.*, 2001). The ages of the sedimentary strata and of the major unconformity in the subsurface beneath Nordmannsfonna is further covered in the discussion.

The figures were designed using GlobalMapper (version 13.00) and CorelDraw (version 2017). Alternative open-source software packages are QGIS and GIMP respectively.

## Results

Two groups of mafic intrusions were identified in eastern Spitsbergen ( Koehl *et al.*, 2022a). The magmatic features that can be directly tied to onshore surface outcrops of Cretaceous dolerite intrusions are presented first as they have implications for the timing of emplacement of the other group of mafic intrusions.

### Cretaceous dolerite dykes and sills of the HALIP

 At the surface, lenticular bodies of moderate- to high-amplitude reflections several kilometers wide (black-shaded in Figure 3a–d) correlate with outcrops of Cretaceous dolerite sills of the HALIP in eastern Spitsbergen (Diabasodden Suite; Dallmann, 2015; Senger *et al.*, 2013). Just below the Cretaceous sills, the 0.5 second (TWT) thick succession of otherwise undisrupted and relatively undeformed, subhorizontal reflections are, in places, crosscut by high-frequency, subvertical disruption surfaces (dotted black lines in Figure 3a–d), e.g., in Domen and Agardhfjellet (see Figure 1d for location). Given their relationship with the Cretaceous sills, these disruptions are interpreted as Cretaceous feeder dykes of the Central Spitsbergen Dolerite Centre ( Nejbert *et al.*, 2011). This is consistent with the respective subvertical and subhorizontal geometries of Cretaceous dykes and sills of the Diabasodden Suite exposed onshore nearby areas (e.g., Senger *et al.*, 2013 their figures 6b and 12). Unlike the moderate–high-amplitude reflections characterizing the dolerite sills, the Cretaceous dolerite feeder dykes appear as low-amplitude disruptions because they are sub-vertical and, thus, the acoustic impedance contrast is minimal along the dykes. Although these subvertical disruptions might possibly be artifacts, their occurrence mostly below Cretaceous sills intruded into post-Mississippian sedimentary rocks suggest they are dykes ( Figure 3a–d).

### West-dipping sills

**Figure 5.**
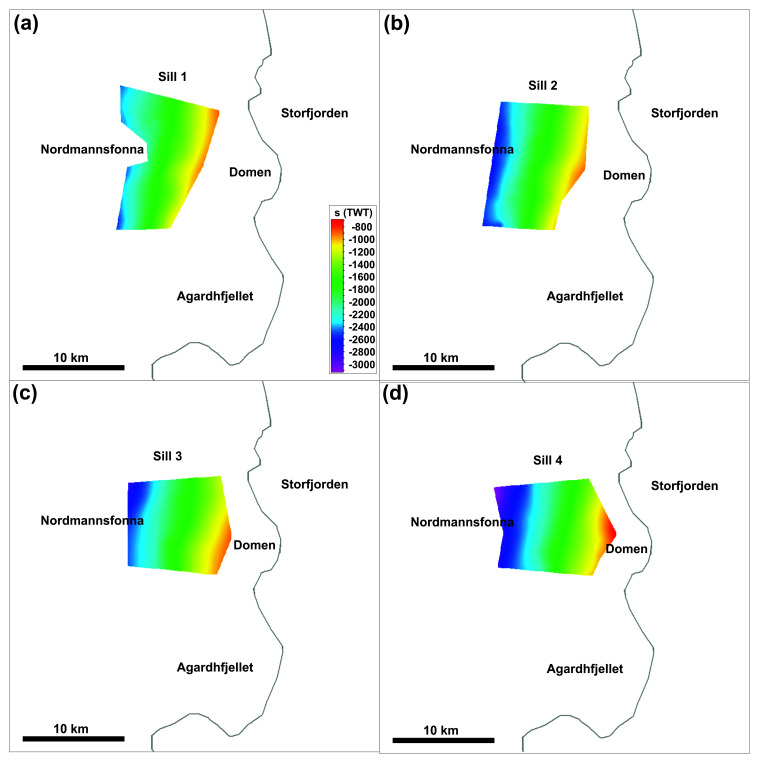
Time maps of the four Middle Devonian–Mississippian sills. The sills are highlighted in fuchsia and numbered 1–4 in Figure 3a–d. The maps delineate the N–S strike and moderately west-dipping geometry of the sills.

 In NNE–SSW along-strike sections, some of the high-amplitude reflections of the second group of intrusions appear overall sub-horizontal and gently undulating and parallel the axis of the NNE-plunging fold of the Kongsfjorden–Cowanodden fault zone ( Figure 3b and d and Figure 5a–d). In E–W cross section, the second group of intrusions is characterized by a swarm of moderately west-dipping, high-amplitude, in places mildly undulating but mostly planar reflections (blue lines in Figure 3a and c). These contrast markedly with the dominantly subvertical low-amplitude or sub-horizontal lenticular character of Cretaceous dolerite dykes and sills respectively (dotted black lines and black bodies in Figure 3b and d). The west-dipping, high-amplitude reflections crosscut but do not offset the Kongsfjorden–Cowanodden fault zone at a c. 120° angle (black lines in Figure 3a–d and Figure 4) and sub-horizontal high- to low-amplitude reflections in the hanging wall of the fault. The latter are interpreted as Devonian–Mississippian sedimentary rocks (yellow lines in orange package in Figure 3a; see Koehl *et al.*, 2022a and Discussion section for further constraints on the age of the strata).

 Upwards, the west-dipping, high-amplitude reflections either terminate within Devonian–Mississippian sedimentary rocks or are truncated by a major erosional unconformity. The reflections are difficult to trace within the Kongsfjorden–Cowanodden fault zone ( Figure 3a and c), which consists of dense mylonite ( Koehl *et al.*, 2022a). However, they show again a high amplitude in the footwall of the fault without offsetting the reflections they crosscut ( Figure 3a and c).

 The lack of offset across the moderately west-dipping, high-amplitude reflections suggests that they correspond to magmatic intrusions. The lower amplitude of these intrusions within denser mylonitic rocks of the Kongsfjorden–Cowanodden fault zone indicates a probable mafic–ultramafic character (i.e., much denser than felsic intrusions), thus presenting lower acoustic impedance contrast with relatively dense mylonites. Seismic depth conversion suggests a moderate dip for the west-dipping intrusions at Nordmannsfonna ( Figure 4). Thus, they are interpreted as sills instead of dykes.

## Discussion

### Timing of pre-Pennsylvanian ultramafic-mafic intrusions in eastern Spitsbergen

The Nordmannsfonna area exhibits flat-lying carbonate-rich sedimentary rocks of the lower Permian Wordiekammen, Gipshuken, and Kapp Starostin formations overlain by lowermost Triassic–Lower Cretaceous sedimentary rocks ( Andresen *et al.*, 1992; Dallmann, 2015; Haremo & Andresen, 1992). These correlate directly with the 0.5 second (TWT) thick succession of sub-horizontal strata seen in seismic data ( Figure 3a–d). Thus, the erosional truncation of the west-dipping intrusions must be Pennsylvanian in age or older, suggesting a pre-Pennsylvanian age for the system of west-dipping ultramafic-mafic sills ( Figure 3a–d).

Previous seismic interpretation of data from Nordmannsfonna suggested the presence of Devonian–Mississippian sedimentary rocks in the hanging wall of a major, folded, late Neoproterozoic thrust system, the Kongsfjorden–Cowanodden fault zone ( Figure 3a–d; Koehl *et al.*, 2022a). A thick (Lower?) Devonian sedimentary succession in the area is supported by the nearby Plurdalen-1 well in southwestern Edgeøya, which penetrated a c. 1000 m-thick succession of red-bed sedimentary rocks that yielded a ca. 410 Ma Rb–Sr age ( Harland & Kelly, 1997, their figure 5.14; see Figure 1b–c for location; see Koehl *et al.*, 2022a for further discussion). Further support is the strong similarity between the Nordmannsfonna portion of the Kongsfjorden–Cowanodden fault zone and the latest Silurian–Devonian core-complex-bounding Keisarhjelmen Detachment in northwestern Spitsbergen ( Braathen *et al.*, 2018; Braathen *et al.*, 2020; Maher *et al.*, 2022). Notable similarities include a north-plunging, pre-Pennsylvanian anticline geometry and km-thick sedimentary successions in the hanging wall of the fault ( Braathen *et al.*, 2018; Figure 3a–d). Thus, a Devonian–Mississippian age is proposed for the ultramafic–mafic sills in Nordmannsfonna.

Since there is no well control on the age of the rock succession in the hanging wall of the Kongsfjorden–Cowanodden fault zone in Nordmannsfonna, it is possible that the west-dipping, pre-Pennsylvanian intrusions are pre-Caledonian in age. Nevertheless, a critical factor in ascribing a Devonian–Mississippian (i.e., post-Caledonian) age to these intrusions is the early–mid Paleozoic reworking of the late Neoproterozoic Kongsfjorden–Cowanodden fault zone. Caledonian tectonism folded the fault into a 15–20 km wide, NNE-plunging anticline, whereas the west-dipping intrusions are only mildly undulating in places ( Figure 3a and c and Figure 5a–d).

This age can be further constrained by considering other Devonian–Mississippian intrusions in central and western Spitsbergen. Examples are basaltic dykes in Ekmanfjorden and Mississippian picritic dykes at Krosspynten and in Purpurdalen, which intrude upper Lower Devonian rocks ( Evdokimov *et al.*, 2006; Sirotkin *et al.*, 2012) of the Dicksonfjorden Member of the Wood Bay Formation ( Blomeier *et al.*, 2003; Dallmann, 2015; Friend *et al.*, 1966; Friend & Moody-Stuart, 1972). The basaltic dykes in Ekmanfjorden are truncated by uppermost Pennsylvanian–lowermost Permian strata of the Wordiekammen Formation ( Figure 2a; Evdokimov *et al.*, 2006; Sirotkin *et al.*, 2012). The sills at Nordmannsfonna are proposed to also intrude rocks of the Dicksonfjorden Member of the Wood Bay Formation because of the widespread distribution of this reddish rock unit in Svalbard ( Dallmann & Piepjohn, 2020; Friend *et al.*, 1966; Friend & Moody-Stuart, 1972; Harland & Kelly, 1997). Hence, a Middle Devonian–Mississippian age is proposed for the ultramafic–mafic sills.

### Model for the development of Devonian-Mississippian intrusions in Svalbard

***Nordmannsfonna***

**Figure 6.**
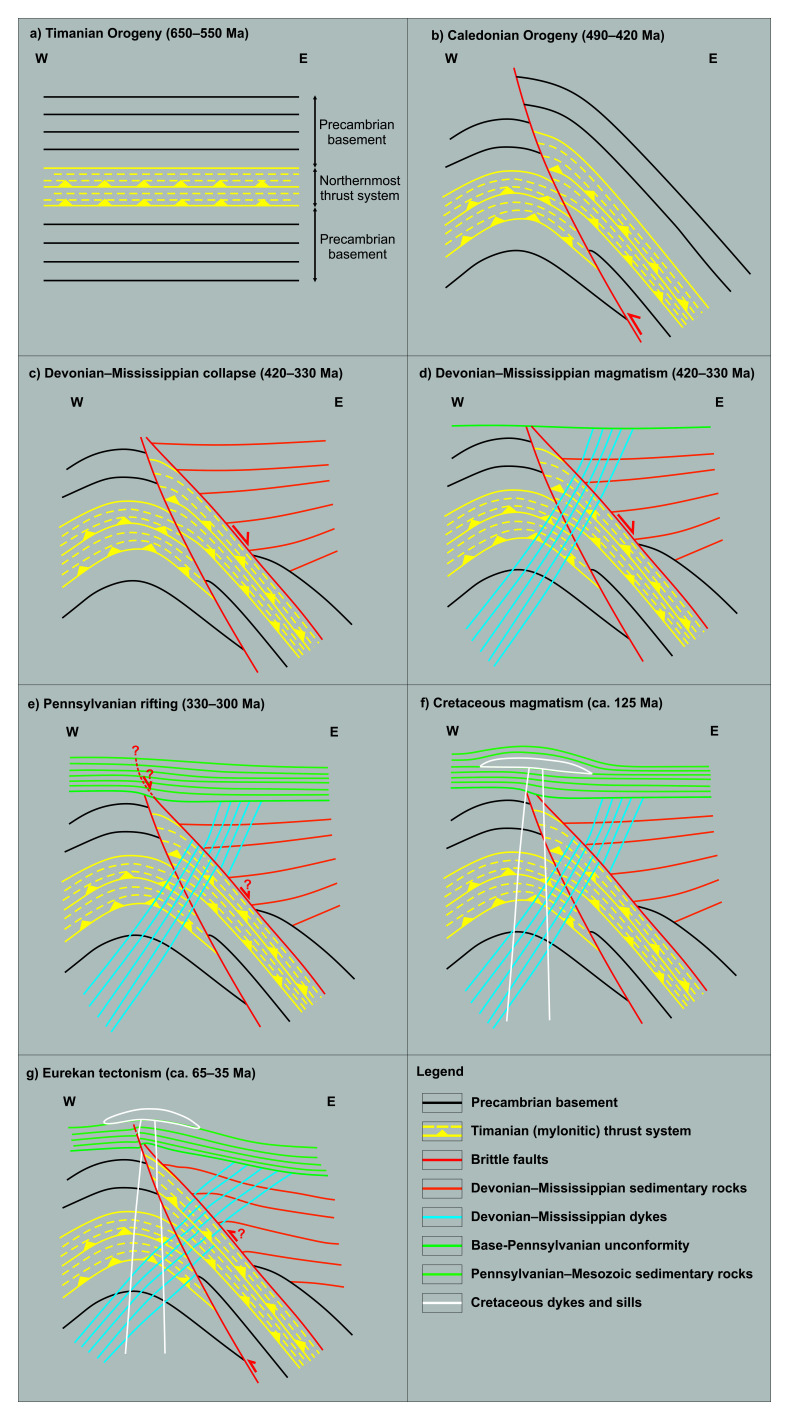
Tectonic history of pre-Pennsylvanian sills at Nordmannsfonna. ( **a**) Formation of a late Neoproterozoic NNE-dipping thrust system (Kongsfjorden–Cowanodden fault zone). ( **b**) Top-west Caledonian thrusting along the east-dipping Agardhbukta Fault and folding of the Kongsfjorden–Cowanodden fault zone. ( **c**) Inversion of the Kongsfjorden–Cowanodden fault zone at the eastern flank of the Caledonian anticline and deposition of syn-tectonic Devonian (–Mississippian?) sedimentary strata during post-Caledonian collapse. ( **d**) Intrusion of west-dipping sills in the Devonian–Mississippian. ( **e**) Regional erosion in the latest Mississippian and deposition of Pennsylvanian sedimentary rocks, along a high-angle splay of the inverted Kongsfjorden–Cowanodden fault zone. ( **f**) Deposition of Mesozoic sedimentary strata and intrusion of Cretaceous dolerite of the HALIP. ( **g**) Partial erosion of Pennsylvanian–Mesozoic strata and minor, early Cenozoic, reactivation of the Kongsfjorden–Cowanodden fault zone and Agardhbukta Fault during the Eurekan tectonic event and mild folding and offset of overlying post-Caledonian sedimentary rocks, intrusions, and base-Pennsylvanian unconformity. Note the back-tilting (i.e., clockwise rotation) of Middle Devonian–Mississippian sills in the hanging wall of the Agardhbukta Fault and of the Kongsfjorden–Cowanodden fault zone. Figure is from Koehl *et al.* (2022a).

Devonian–Mississippian sills in Nordmannsfonna truncate the late Neoproterozoic, NNE-dipping Kongsfjorden–Cowanodden fault zone ( Figure 6a), which was folded and reactivated and/or overprinted during the Caledonian Orogeny ( Figure 6b). Devonian–Mississippian inversion of the fault is suggested by the presence of Devonian (to Mississippian?) sedimentary rocks in the hanging wall of the fault in the east ( Figure 3a and c and Figure 6c; Koehl *et al.*, 2022a). The sills intruded at a c. 60° angle to the Kongsfjorden–Cowanodden fault zone, possibly along extensional fractures (tension gashes? Beach, 1975) formed through reactivation and/or overprinting of the fault during late–post-Caledonian extensional collapse ( Figure 6d). Magma intruded where the thrust system was thinnest ( Figure 6c–d). Despite the geometrical and seismic facies similarities of the intrusions in Nordmannsfonna and Pennsylvanian–Permian intrusions of the Skagerrak LIP in southern Norway ( Phillips *et al.*, 2018a) normal-fault-block tilting is not considered because of the lack of tilting of sedimentary strata in the hanging wall of the Kongsfjorden–Cowanodden fault zone ( Figure 3a and c). Devonian–Mississippian magma emplacement was followed by deposition of Pennsylvanian to Mesozoic sedimentary strata, intrusion of Early Cretaceous sills and dykes of the HALIP, and mild Eurekan folding and reactivation of the Kongsfjorden–Cowanodden fault zone ( Figure 6e–g Koehl *et al.*, 2022a).

***Ekmanfjorden***

**Figure 7.**
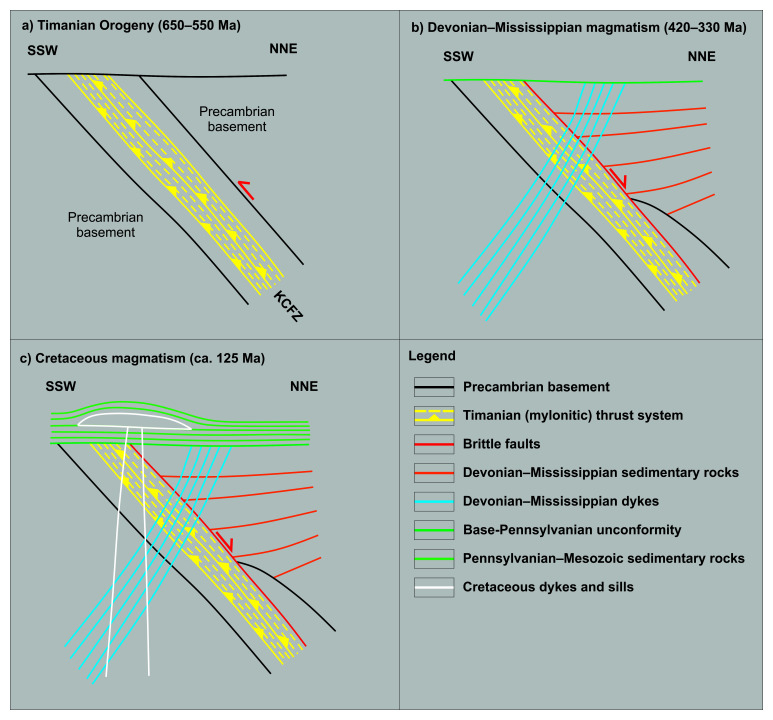
Tectonic history of Middle Devonian–Mississippian (–earliest Late Pennsylvanian?) basaltic dykes in Ekmanfjorden. ( **a**) Formation of the NNE-dipping Kongsfjorden–Cowanodden fault zone (KCFZ) during the Timanian Orogeny in the late Neoproterozoic. ( **b**) Deposition of several kilometers thick, Lower Devonian, collapse-related sedimentary rocks of the Wood Bay Formation along the inverted Kongsfjorden–Cowanodden fault zone and intrusion of Middle Devonian–Mississippian dykes sub-perpendicular to the fault. ( **c**) Deposition of Mesozoic sedimentary strata and intrusion of Cretaceous dolerite of the HALIP along a WNW–ESE-trending axis, i.e., parallel to the fault. Notice that in this case, the Eurekan event possibly led to mild (km-scale) sinistral strike-slip movements along the Kongsfjorden–Cowanodden fault zone, which are not relevant to the intrusion of the dykes in Ekmanfjorden.

The situation in Ekmanfjorden ( Figure 1c) is similar to that described for the intrusions in Nordmannsfonna. The main difference is the strike of the intrusions, which is WNW–ESE (320–330°) with a steep dip (60–70°) to the southwest ( Figure 2a). This is probably due to the NNE-dipping geometry of the Kongsfjorden–Cowanodden fault zone in this area, i.e., absence of Caledonian reworking ( Figure 7a). As in Nordmannsfonna, the dykes intruded at a 60° angle to the fault, most likely along extensional fractures (tension gashes?) formed during extensional reactivation of mylonitic thrust surfaces during late–post-Caledonian extensional collapse ( Figure 7b). Cretaceous intrusions of the HALIP also intruded along a WNW–ESE-striking axis (e.g., Hatten feeder dyke; Senger *et al.*, 2013), i.e., likely along the Kongsfjorden–Cowanodden fault zone ( Figure 7c).

### Possible relationship to diamond-rich intrusions in northwestern Russia

Kimberlitic accessory minerals (e.g., chromium spinel, chromium diopside, moissanite, and pyrope) in the dykes at Krosspynten and Purpurdalen ( Evdokimov *et al.*, 2006; Sirotkin *et al.*, 2012) suggest that Middle Devonian–Mississippian intrusions in Svalbard might have a composition similar to that of kimberlitic dyke swarms of the Kola–Dnieper LIP in northwestern Russia ( Larionova *et al.*, 2016; Puchkov *et al.*, 2016; Terekhov *et al.*, 2012). Considering the overlapping time constraints for emplacement of the sills in Nordmannsfonna (present study; Figure 3a–d) and in Systertoppane and Ragnarbreen ( Teben’kov *et al.*, 1996; Figure 1c), it is possible that these too may have a composition similar to that of the dyke swarms in northwestern Russia.

This inference is supported by evidence from Upper Triassic sandstones of the De Geerdalen Formation in Svalbard, which contain detrital chromium spinel grains compositionally similar to spinel group minerals from kimberlites and related rocks ( Svånå Harstad, 2016, his figures 6.48 and 6.49). Based on the absence of Triassic magmatic rocks in Svalbard, Svånå Harstad (2016) argued that the chromium spinel in the De Geerdalen Formation in Edgeøya, Hopen, and central–southern Spitsbergen originated from Lower Triassic Siberian Trap ultramafic magmatic rocks in northern Russia. Such an origin is plausible given the age peak at ca. 250 Ma for detrital zircon in the De Geerdalen Formation in Svalbard ( Bue & Andresen, 2014, their Figure 6). The Siberian flood volcanic province in northern Russia comprises numerous alkaline–ultramafic complexes, and kimberlitic to lamprophyric rocks of these minor intrusions could be a source of detrital chromium-rich spinel (e.g., Augland *et al.*, 2019; Reguir *et al.*, 2021; Sun *et al.*, 2014). However, Triassic kimberlite magmatism is also known from northern Canada ( Tappe *et al.*, 2018; Zurevinski *et al.*, 2008) and cannot be excluded as a source of detrital chromium-spinel.

Regardless, spinel group minerals are sensitive to oxidization-related alteration and therefore do not typically travel as far from their magmatic sources as other heavy minerals such as zircon and garnet ( Dare *et al.*, 2016; Griffin *et al.*, 1997; Haugaard *et al.*, 2021). Thus, a proximal source for chromium spinel in the Upper Triassic De Geerdalen Formation is more likely than magmatic rocks in northern Russia or Canada. In addition, the distance between Svalbard and the Siberian Traps or Arctic Canada is rather large, which suggests that detrital Siberian spinel would occur in fine-grained, clay-dominated sedimentary rocks similar to the Ural-sourced Triassic formations in the Norwegian sector of the Barents Sea, which are the offshore time-equivalent of the De Geerdalen Formation (e.g., Snadd Formation; Seim *et al.*, 1988; Smelror *et al.*, 2009).

Since chromium spinel was found in sandstone (i.e., relatively coarse-grained sedimentary rocks), the sediment source is likely proximal to the depocenter (e.g., McLaren & Bowles, 1985). Thus, it is probable that the detrital chromium spinel in the De Geerdalen Formation derived from nearby magmatic rocks in Svalbard, e.g., Devonian–Mississippian dykes of central ( Evdokimov *et al.*, 2006) and eastern Spitsbergen (present study; Figure 3a–d). Since they potentially have a composition similar to kimberlitic and ultramafic-alkaline magmatism in northwestern Russia such as on the Kola Peninsula ( Puchkov *et al.*, 2016), additional magmatic sources of the detrital chromium spinel population could be the ultramafic–mafic dykes in Nordaustlandet ( Krasil’scikov, 1973; Ohta, 1982), and/or primitive magmatic rocks from Loppa High (and other basement highs) in the Barents Sea ( Norsk Hydro, 1986). This is supported by U–Pb ages of detrital zircons suggesting that sediments of the De Geerdalen Formation partly derived from Devonian–Mississippian sedimentary rocks on a second or third cycle ( Bue & Andresen, 2014, their figure 6) and by the erosional truncation of Devonian–Mississippian dykes in Nordmannsfonna ( Figure 3a and c).

Another possible line of evidence for a Devonian–Mississippian origin of the detrital chromium spinel grains is the absence of rocks of the Upper Triassic De Geerdalen Formation in Nordmannsfonna and nearby areas, which suggests that the area was subjected to erosion during the Middle–Late Triassic. This is further supported by paleosols ( Enga, 2015; Lord *et al.*, 2022) and shallow marine to terrestrial sedimentary rocks ( Johansen, 2016) in the De Geerdalen Formation onshore Edgeøya and in the Snadd Formation in the Barents Sea. These suggest an environment prone to erosion during the Late Triassic evolution of Svalbard and surrounding areas.

Coal fragments were found in the Middle–Upper Triassic Barentsøya, Tschermakfjellet, De Geerdalen and Wilhelmsøya formations in exploration well 7816/12-1 in Reindalen ( Eide *et al.*, 1991; see location in Figure 1c). Coal also occurs in the southeastern Barents Sea ( Smelror *et al.*, 2009) but the fragility of coal makes it unlikely to have been transported as large (centimeter-sized) fragments over long distances, suggesting a nearby source. Since no autochthonous coal has been reported from the Triassic sedimentary rocks of Svalbard and nearby areas of the Barents Sea ( Dallmann *et al.*, 1999; Smelror *et al.*, 2009), the presence of coal fragments in Triassic sedimentary rocks suggests reworking and redeposition of Mississippian coal measures.

Middle Devonian–Mississippian sills in Nordmannsfonna are presently overlain by a c. 1.5 km thick succession of Lower Pennsylvanian–Mesozoic sedimentary rocks ( Figure 4), including the Malte Brunfjellet Formation ( Scheibner *et al.*, 2015) and the Wordiekammen, Gipshuken, and Kapp Starostin formations ( Dallmann *et al.*, 1999; Dallmann, 2015). This suggests that the sills were likely eroded during the Late Mississippian–earliest Pennsylvanian but were subsequently buried below Lower Pennsylvanian–Middle Triassic sedimentary rocks, including deposition of the De Geerdalen Formation during the Late Triassic. Nevertheless, the abundant Devonian–Mississippian zircons in sandstones of the De Geerdalen Formation ( Bue & Andresen, 2014) suggests that Devonian–Mississippian igneous rocks were exposed nearby during the Late Triassic. A possible source is the Loppa High, where potential Mississippian intrusions were penetrated by exploration wells ( Norsk Hydro, 1986). There, exploration well 7120/2-1 penetrated dolerite intrusions, which yielded an inconclusive, probably biased 179 Ma (latest Early Jurassic) K–Ar age, which should be treated as a minimum ( https://factpages.sodir.no/en/wellbore/PageView/Exploration/All/473; Norsk Hydro, 1986). The dolerites are unconformably covered by sedimentary rocks equivalent to those of the latest Mississippian Landnøringsvika Formation in Bjørnøya and Hultberget Formation in Spitsbergen ( Norsk Hydro, 1986). The Loppa High is a long-lived basement high, which is cored by a folded late Neoproterozoic thrust system ( Koehl *et al.*, 2023c) and was repeatedly eroded during the Paleozoic–Mesozoic ( Indrevær *et al.*, 2017 their figure 5a; Anell *et al.*, 2019; Koehl *et al.*, 2023c their supplement S2).

The sources of Triassic sediments in Svalbard are still a matter of debate and some studies argue for a dominant (south-) easterly source (e.g., the Urals; Anell *et al.*, 2014; Gilmullina *et al.*, 2021; Smelror *et al.*, 2009). Others suggest sourcing from the (north-) west (Laurentia; Bue & Andresen, 2014). Bue and Andresen (2014) argue that Early–Middle Triassic sediments in Svalbard were sourced from northern Greenland and then reworked and redeposited during the Late Triassic as suggested by the mixing of Early–Middle Triassic zircons with Late Triassic zircons in Upper Triassic rocks of the De Geerdalen Formation. This model is further supported by rare specimens of Leschikisporis aduncus spores in Upper Triassic strata of the De Geerdalen Formation on Hopen, which are abundant in the underlying Lower–Middle Triassic sedimentary rocks and therefore considered to be reworked ( Paterson & Mangerud, 2015). Sourcing Lower–Middle Triassic sediments from the (north-) west and their reworking during the Late Triassic is therefore plausible. It would have allowed transport of chromium spinel grains eroded from the Middle Devonian–Mississippian ultramafic–mafic intrusions in Nordmannsfonna or nearby areas towards the (south-) east, e.g., towards Edgeøya where the chromium spinel was found ( Svånå Harstad, 2016).

### A devonian–mississippian large igneous province in arctic Norway and Russia

The sills at Nordmansfonna and those previously identified at Krosspynten, Purpurdalen, and Ekmanfjorden ( Evdokimov *et al.*, 2006; Gayer *et al.*, 1966; Sirotkin *et al.*, 2012) and Systertoppane and Ragnarbreen ( Teben’kov *et al.*, 1996; Figure 1c) suggest an episode of widespread mantle-derived magmatism in Svalbard during the Middle Devonian–Mississippian. Extensive magmatism in Svalbard at that time is supported by the Mississippian age obtained for a metadolerite in southern Spitsbergen ( Birkenmajer *et al.*, 2010).

Indirect evidence for widespread Late Devonian–Mississippian magmatism in Svalbard comes from the 377–326 Ma ^40^Ar– ^39^Ar ages for micaschists and mylonite in Oscar II Land in western Spitsbergen, which suggest a post-Caledonian thermal event ( Michalski *et al.*, 2017). Since the Svalbardian event (presumed Late Devonian–earliest Mississippian E–W-oriented contraction) is unlikely to have occurred on Svalbard ( Koehl *et al.*, 2022b), the thermal event recorded by these Late Devonian–Mississippian ^40^Ar– ^39^Ar ages may be regional magmatism possibly related to late–post-Caledonian extensional collapse and/or rifting. Note that the latest Devonian–earliest Mississippian ages for amphibolite-facies metamorphism on Prins Karls Forland ( Kosminska *et al.*, 2020) are associated with extensional kinematics ( Schneider *et al.*, 2018), i.e., with the collapse of the Caledonides ( Koehl *et al.*, 2022b).

A Mississippian age was recently obtained for dolerite dykes in exploration well 7120/2-1 (see completion report at www.sodir.no) on Loppa High in the western Barents Sea ( Norsk Hydro, 1986). Extensive Devonian–Mississippian magmatism in the western Barents Sea region is supported by abundant ignimbrite and basalt in sidewall cores at 2505–3502 m depth within (Upper Devonian–?) Mississippian sedimentary rocks in well 7120/2-1 (see completion report at www.sodir.no).

Analogous Mississippian dolerite dykes are known from northern Finnmark in northern Norway ( Lippard & Prestvik, 1995; Lippard & Prestvik, 1997; Roberts *et al.*, 1991). Late Devonian dykes occur in eastern Finnmark ( Guise & Roberts, 2002), on the Kola Peninsula, the Arkhangelsk Province ( Larionova *et al.*, 2016; Roberts & Onstott, 1995; Terekhov *et al.*, 2012), and in the Ural Mountains and the Pay–Khoy Ridge on the Yugorsky Peninsula in northwestern Russia ( Puchkov *et al.*, 2016; Shaybekov, 2010).

Puchkov *et al.* (2016) postulated a Devonian plume center in the southeastern Barents Sea at the center of a multi-armed Devonian paleorift ( Figure 1a–b). A NE–SW-trending arm extends from the southeastern Barents Sea to the Kola Peninsula, where it continues as the Kontozero Graben ( Kramm *et al.*, 1993) hosting diamond-rich kimberlitic dyke swarms of Devonian age ( Larionova *et al.*, 2016; Terekhov *et al.*, 2012; Figure 1b). Another rift segment strikes WNW–ESE and extends from the southeastern Barents Sea to the Pechora Basin and the Yugorsky Peninsula in the southeast. There it comprises a major, hundreds of km-long Devonian dolerite dyke swarm ( Puchkov *et al.*, 2016; Shaybekov, 2010; Figure 1a–b). Yet another WNW–ESE-striking arm extends from the rift’s center in the southeastern Barents Sea to eastern Svalbard, near the location of the newly reported sills in Nordmannsfonna ( Figure 1a–b). Thus, it is conceivable that the mantle plume center inferred by Puchkov *et al.* (2016) generated the Devonian dykes on the Kola Peninsula ( Terekhov *et al.*, 2012) as well as the Middle Devonian–Mississippian sills in eastern Spitsbergen ( Figure 3a–d) and dykes in central ( Evdokimov *et al.*, 2006; Gayer *et al.*, 1966; Sirotkin *et al.*, 2012; Teben’kov *et al.*, 1996) and southern Spitsbergen ( Birkenmajer *et al.*, 2010).

The newly identified intrusions in Nordmannsfonna and those already known in Svalbard ( Evdokimov *et al.*, 2006; Gayer *et al.*, 1966; Sirotkin *et al.*, 2012), as well as potential analogs from the Loppa High ( Norsk Hydro, 1986), suggest that the Middle Devonian–Mississippian Kola–Dnieper LIP extends all the way to Svalbard and the western Barents Sea. The revised LIP thus extends c. 2000 km along a WNW–ESE-trending axis from the Yugorsky Peninsula in northwestern Russia to Svalbard and over 3200 km from the Pripyat–Dnieper–Donets Rift to Svalbard along a N–S-trending axis ( Figure 1a). This would require intense plume-related magmatism to have occurred thousands of kilometers from the presumed plume center in the southeastern Barents Sea ( Puchkov *et al.*, 2016; Figure 1a–b). The west-dipping character of the newly reported ultramafic-mafic sills from eastern Spitsbergen ( Figure 3a–d, Figure 4, and Figure 5a–d) is inconsistent with these magmatic bodies forming part of a giant radiating dyke swarm sourced from a plume center in the southeastern Barents Sea ( Puchkov *et al.*, 2016; Figure 1a–b). The observations argue instead for a local lithospheric mantle source beneath Svalbard, e.g., due to mantle decompression during late–post-Caledonian extensional collapse.

### Devonian–Mississippian magmatism in Baltica and Siberia and implications radiating dyke swarms

Magmatism contemporaneous with the Kola–Dnieper LIP occurred in the Yakutsk–Vilyui LIP during the Late Devonian–Mississippian ( Courtillot *et al.*, 2010; Hamilton *et al.*, 2004; Powerman *et al.*, 2013; Ricci *et al.*, 2013; Tretiakova *et al.*, 2017) and in the Altay–Sayan LIP during the Early Devonian–Mississippian ( Babin *et al.*, 2004; Broussolle *et al.*, 2019; Kong *et al.*, 2023; Kuzmin *et al.*, 2010; Malkovets *et al.*, 2003; Predtechensky & Krasnov, 1967; Safonova *et al.*, 2004; Vorontsov *et al.*, 2013; Vorontsov *et al.*, 2021).

The strong similarities between the Kola–Dnieper LIP and the Yakutsk–Vilyui LIP, including their (extensional) structural styles, suggest a similar setting of emplacement. More specifically, both LIPs are characterized by multi-arm paleorifts and numerous magmatic bodies have kimberlitic and alkaline mafic to ultramafic compositions. Dyke swarms seem to radiate from potential plume centers in the southeastern Barents Sea and southern Vilyui Rift respectively ( Figure 1a). Some studies suggested that both LIPs originated from mantle plumes sourced from the edge of the African Large Low-Shear Velocity Province ( Puchkov *et al.*, 2016). This model relies on Large Low-Shear Velocity Provinces being stationary and underlying the appropriate regions and on accurate plate reconstructions back to Paleozoic times. The model is also based on the converging pattern displayed by giant radiating dyke swarms, from which magmatic plume centers are commonly inferred ( Ernst *et al.*, 1995; Ernst & Buchan, 1997; Minakov *et al.*, 2017; Puchkov *et al.*, 2016).

 Similarities in magmatism of the Kola–Dnieper LIP and the Altay–Sayan LIP are more tenuous. Notably, the timing of the first phase of magmatism (Early–Middle Devonian) only partly overlaps with the Kola–Dnieper LIP ( Babin *et al.*, 2004; Malkovets *et al.*, 2003; Predtechensky & Krasnov, 1967). In addition, the Altay–Sayan LIP consists dominantly of granites with very few mafic–ultramafic rocks ( Predtechensky & Krasnov, 1967; Safonova *et al.*, 2004; Vorontsov *et al.*, 2013), which contrasts with the dominance of mafic–ultramafic intrusions in the Kola–Dnieper LIP ( Larionova *et al.*, 2016; Mahotkin *et al.*, 2000; Pease *et al.*, 2016; Puchkov *et al.*, 2016; Terekhov *et al.*, 2012; Wilson *et al.*, 1999). Nevertheless, some studies suggest an intermediate composition between intraplate (ocean-island-basalts-like) and active margin (island-arc-basalts-like) magmatism. Consequently, the influence of a mantle plume on the Devonian–Mississippian magmatism recorded by the Altay–Sayan LIP was suggested (e.g., Kuzmin *et al.*, 2010; Vorontsov *et al.*, 2013; Vorontsov *et al.*, 2021), possibly the same plume proposed to be the source of the Yakutsk–Vilyui LIP ( Kuzmin *et al.*, 2010).

In the study area ( Figure 1d) and elsewhere in Spitsbergen ( Figure 1c), the intrusion of Middle Devonian–Mississippian mantle-derived magmatic rocks is probably related to late–post-Caledonian, collapse-related reactivation–overprinting along preexisting Timanian thrust systems ( Figure 6 and Figure 7). This line of reasoning may be applied to the mafic–ultramafic intrusions of the entire Kola–Dnieper LIP ( Figure 1a). In northeastern Norway ( Figure 1b), WNW–ESE- and NE–SW-striking Late Devonian–Mississippian dolerite dykes intruded respectively parallel to Timanian thrusts and along bedding surfaces, the latter of which were folded into tight, northwest-plunging folds during the Caledonian Orogeny ( Koehl *et al.*, 2019; Koehl & Stokmo, 2024; Nasuti *et al.*, 2015).

On the Kola Peninsula ( Figure 1a–b), NE–SW-striking ultramafic–mafic dykes and the related Kontozero Graben formed parallel to preexisting Caledonian grain ( Koehl *et al.*, 2022a their figure 5 showing Caledonian grain extending onto the Kola Peninsula). Finally, in the Urals, Kanin Peninsula, Pechora Basin, Yugorsky Peninsula, and Novaya Zemlya ( Figure 1a–b), Devonian–Mississippian intrusions follow inherited Timanian thrust systems ( Korago *et al.*, 2004; Kostyuchenko *et al.*, 2006; Lopatin *et al.*, 2001; Lorenz *et al.*, 2004; Olovyanishnikov *et al.*, 2000; Figure 1b). Thus, the existence of a mantle plume in the Barents Sea is not required to explain the radiating pattern of Devonian–Mississippian dyke swarms in this region.

In eastern Europe, the Pripyat–Dnieper–Donets Rift developed parallel to the Tornquist Zone, which is a long-lived zone of deformation and magmatism along the southwestern edge of the East European Craton ( Abramovitz *et al.*, 1999; Cotte *et al.*, 2002; Coward, 1990; Graversen, 2009; Hossein Shomali *et al.*, 2006; Narkiewicz *et al.*, 2015; Pegrum, 1984; Phillips *et al.*, 2018b; Tappe *et al.*, 2016). Evidence for late Neoproterozoic deformation along the Tornquist Zone is preserved in exotic Gondwanan terranes in the southwest ( Belka *et al.*, 2003; Narkiewicz & Petecki, 2017; Zelazniewicz *et al.* 2009). However, this major structure may be as old as Paleoproterozoic ( Koehl *et al.*, 2024). Thus, in this case too, a mantle plume is not required to explain the geometry of the Pripyat–Dnieper–Donets Rift and dyke swarms in the Urals, which all appear to radiate from the Caspian Sea in map view, but most likely emplaced along preexisting late Neoproterozoic (or older) orogenic structures.

A similar approach can be applied to the Yakutsk–Vilyui and Altay–Sayan LIPs, whose emplacement may have been influenced by pre-Devonian orogenic structures. Possible candidates to have facilitated intrusion of magma of the Altay–Sayan LIP are orogenic structures of the late Neoproterozoic–early Paleozoic Central Asian Orogenic Belt/Baikalian Orogen, which occur on the southern rim of the Siberian Craton and largely overlap with the location of the Altay–Sayan LIP ( Filatova & Khain, 2008; Lehman *et al.*, 2010; Wang *et al.*, 2021).

In eastern Siberia, the NE–SW-striking southwestern dyke swarm of the Yakutsk–Vilyui LIP coincides with and runs parallel to structures of the Akitkan Orogen ( Didenko *et al.*, 2009; Donskaya *et al.*, 2009; Donskaya, 2020), which extends from the Baikal Lake to the elbow-shaped corner of the Verkhoyansk Mountains where a potential plume center was proposed ( Figure 1a). Farther north, the northwestern dyke swarm of the Yakutsk–Vilyui LIP strikes NW–SE, i.e., parallel to overlapping Paleoproterozoic shear zones and deformation belts of the Hapschan Orogen/Khapchan Belt, which separates the Anabar Province from the Olenek Province ( Donskaya, 2020; Gusev *et al.*, 2021; Sears & Price, 2000). Thus, the radiating pattern of dykes of the Yakutsk–Vilyui LIP is most likely related to inherited Paleoproterozoic orogenic structures rather than to radial uplift around a mantle plume center in eastern Siberia.

Other examples of radiating dyke swarms are the Early Cretaceous dykes related to the HALIP in Svalbard, Franz Josef Land, and Arctic Canada, which appear to converge in the Arctic Ocean north of Franz Josef Land and Svalbard ( Figure 1a). In Franz Josef Land, Cretaceous dolerite intrusions strike WNW–ESE ( Minakov *et al.*, 2017; Shipilov & Karyakin, 2014; Figure 1a). In Svalbard, they strike N–S in the northeast and the southwest, and WNW–ESE in central Spitsbergen ( Dallmann, 2015; Grogan *et al.*, 2000; Maher, 2001; Nejbert *et al.*, 2011; Senger *et al.*, 2013), i.e., parallel to well-known Caledonian fabrics and structures in northeastern (e.g., Dumais & Brönner, 2020; Flood *et al.*, 1969; Gee *et al.*, 1994; Johansson *et al.*, 2004; Johansson *et al.*, 2005; Ohta, 1982; Witt-Nilsson *et al.*, 1998) and southwestern Svalbard ( Birkenmajer, 1975; Birkenmajer, 2004; Manby, 1986) and to Timanian thrust systems in central Spitsbergen ( Koehl, 2020; Koehl, 2021; Koehl *et al.*, 2022a; Koehl *et al.*, 2023b). In Arctic Canada, Early Cretaceous dolerite dykes strike NE–SW from Melville Island to Ellesmere Island and N–S in Axel Heiberg Island ( Ernst & Buchan, 1997; Jackson & Halls, 1988; Kingsbury *et al.*, 2016; Figure 1) and are parallel to structures formed during the M'Clintock Orogeny ( Estrada *et al.*, 2018; Powell & Schneider, 2022).

Mantle plume models are by default widely applied to explain LIPs worldwide (e.g., Burke & Torsvik, 2004; Ernst, 2014; Ernst *et al.*, 2019). However, geochemical data do not necessarily require deep origins and constraints from magmatic rocks remain inconclusive as to whether upwelling mantle plume sources produced certain LIPs ( Hawkesworth & Schersten, 2007; Tappe *et al.*, 2020). Thus far, structural evidence such as giant radiating dyke swarms have been assumed to indicate mantle plume activity (e.g., Ernst *et al.*, 1995; Ernst & Buchan, 1997; Minakov *et al.*, 2017; Puchkov *et al.*, 2016). The present work shows, however, that the apparent radiating pattern of some groups of intrusions extending hundreds to thousands of km is merely controlled by preexisting orogenic structures (e.g., Kola–Dnieper LIP, Yakutsk–Vilyui LIP, and HALIP). As such, even if the magma was sourced from a plume center, the apparently radiating pattern of these groups of intrusions is not the product of plume-related uplift as defined in Ernst *et al.* (1995). Thus, interpretation of intrusions as radiating swarms and their use in supporting mantle plumes must be evaluated on an individual basis.

## Conclusions

1) Abundant Middle Devonian–Mississippian sills occur in the subsurface of eastern Svalbard.
2) These sills probably intruded along extensional fractures in reactivated–overprinted portions of a major late Neoproterozoic (Timanian) thrust system, the Kongsfjorden–Cowanodden fault zone, during late–post-Caledonian, collapse-related extension.
3) Middle Devonian–Mississippian intrusions in Svalbard show affinities with and were likely emplaced in a similar extensional setting as ultramafic–mafic intrusions in northwestern Russia, as suggested by kimberlitic accessory minerals and the chemical composition of chromium spinel in Upper Triassic sedimentary rocks in eastern Svalbard.
4) The Middle Devonian–Mississippian ultramafic–mafic intrusions in Svalbard may represent part of a vast regional magmatic province that extends from the Ural Mountains in the east to Svalbard in the northwest, and possibly to the Pripyat–Dnieper–Donets Rift in the southwest.
5) This study suggests that the radiating patterns of ultramafic–mafic dyke swarms in late–post-orogenic extensional provinces, rifts, and Large Igneous Provinces can be controlled by inherited orogenic structures and are therefore not necessarily the product of plume-related uplift.

## Data Availability

The data is public (CC-0) and may be accessed from the Norwegian National Data Repository for Petroleum Data (DISKOS repository) by contacting the Norwegian Offshore Directorate by email at https://www.sodir.no/en/diskos/.
