# Supplementary material for: Devonian–Mississippian magmatism related to extensional collapse in Svalbard: implications for radiating dyke swarms
Source: Open Res Eur. 2024 Dec 12;4:262. [Version 1] doi: 10.12688/openreseurope.18798.1 (PMC11757928; doi:10.12688/openreseurope.18798.1)
Supplement: Peer review history

# Reviewer report: Reviewer response for version 1

**DOI:** 10.21956/openreseurope.20341.r49580

**Published:** 2025-01-23

**Authors:** Alexei Ivanov

**Affiliations:** 1 Russian Academy of Sciences, Moscow, Russian Federation

The paper by Koehl et al. "Devonian-Mississippian magmatism related to extensional collapse in Svalbard: implications for radiating dyke swarms" is devoted to the identification of intrusive rocks in East Spitsbergen using seismic data and the association of these data with geological maps. The authors attempt (successfully) to identify Devonian intrusive rocks and draw some general geodynamic implications. In my opinion, the paper is of good quality and can be published after minor revisions. See comments below.

1. Fig. 1. Panel d is not indicated as d.
2. Little is known about the western extent of the paleorift – Svalbard is located to north not west. Please rephrase.
3. Ultramafic–mafic rocks such as alkaline basalts dominate - alkaline basalts are not ultramafic. Please rephrase.
4. On the Kola Peninsula, it includes ultramafic-carbonatite complexes of the Kola Alkaline Province (Downes et al., 2005) – one reference here is probably not enough. At least, credit to Kogarko, 1987 [Ref 1] should be made.
5. The Early Devonian age is probably a minimum age as suggested by excess argon in the sample. – what is the evidence for excess argon?

General comment: Not cited paper by Burzyński et al., 2018 [Ref 2] could be of interest.

Is the study design appropriate and does the work have academic merit?

Yes

Is the work clearly and accurately presented and does it cite the current literature?

Yes

If applicable, is the statistical analysis and its interpretation appropriate?

Not applicable

Are all the source data underlying the results available to ensure full reproducibility?

Yes

Are the conclusions drawn adequately supported by the results?

Yes

Are sufficient details of methods and analysis provided to allow replication by others?

Yes

Reviewer Expertise:

Igneous petrology, geochemistry, geochronology

I confirm that I have read this submission and believe that I have an appropriate level of expertise to confirm that it is of an acceptable scientific standard.

## References

[1] : Alkaline rocks of the eastern part of the Baltic Shield (Kola Peninsula). *Geological Society, London, Special Publications* .1987;30(1) : 10.1144/GSL.SP.1987.030.01.26 531-544 10.1144/GSL.SP.1987.030.01.26

[2] : Mineralogical, Rock-Magnetic and Palaeomagnetic Properties of Metadolerites from Central Western Svalbard. *Minerals* .2018;8(7) : 10.3390/min8070279 10.3390/min8070279

# Reviewer report: Reviewer response for version 1

**DOI:** 10.21956/openreseurope.20341.r48634

**Published:** 2025-01-15

**Authors:** Natasha Stanton

**Affiliations:** 1 University of Rio de Janeiro State, Rio de Janeiro, RJ, Brazil

The authors present an article on the Devonian-Missipian magmatism at Svalbard and Discuss its genesis and correlations with the regional LIP of the High Arctic. The article covers a very relevant subject in Plate Tectonics, presenting high quality data and very interesting results that clearly show the interplay between tectonics and magmatism through successive deformational Periods. 

 The results point out the direct correlation between the basement orogenic structures and the mafic-ultramafic intrusions, showing the impact of preexisting thrusts, and fault zones on the emplacement of sills and dykes, specially on the focusing pathways for the magmatism.

 The authors present a complete review of the existing plume hypotheses for the origin of radiating dyke swarms, showing that in most cases the structural inheritance constitutes the best explanation for the dykes and other intrusives formation. The observations are well presented and the interpretations are original. The study is a great contribution to our understanding of the mechanisms of magma emplacement, specially in the context of the plate tectonic cycles, and for the knowledge of the Svalbard.

Is the study design appropriate and does the work have academic merit?

Yes

Is the work clearly and accurately presented and does it cite the current literature?

Yes

If applicable, is the statistical analysis and its interpretation appropriate?

Not applicable

Are all the source data underlying the results available to ensure full reproducibility?

Yes

Are the conclusions drawn adequately supported by the results?

Yes

Are sufficient details of methods and analysis provided to allow replication by others?

Yes

Reviewer Expertise:

Marine Geophysics applied to geotectonics

I confirm that I have read this submission and believe that I have an appropriate level of expertise to confirm that it is of an acceptable scientific standard.
